# A 5‐facet framework to describe patient engagement in patient safety

**DOI:** 10.1111/hex.12815

**Published:** 2018-08-29

**Authors:** Lenora Duhn, Jennifer Medves

**Affiliations:** ^1^ School of Nursing Queen's University Kingston Ontario Canada

**Keywords:** community hospital, patient engagement, patient safety, qualitative study

## Abstract

**Background:**

Health care remains unacceptably error prone. Recently, efforts to address this problem have included the patient and their family as partners with providers in harm prevention. Policymakers and clinicians have created patient safety strategies to encourage patient engagement, yet they have typically not included patient perspectives in their development or been comprehensively evaluated. We do not have a good understanding of “if” and “how” patients want involvement in patient safety during clinical interactions.

**Objective:**

The objective of this study was to gain insight into patients’ perspectives about their knowledge, comfort level and behaviours in promoting their safety while receiving health care in hospital.

**Methods:**

The study design was a descriptive, exploratory qualitative approach to inductively examine how adult patients in a community hospital describe health‐care safety and see their role in preventing error.

**Results:**

The findings, which included participation of 30 patients and four family members, indicate that although there are shared themes that influence a patient's engagement in safety, beliefs about involvement and actions taken are varied. Five conceptual themes emerged from their narratives: Personal Capacity, Experiential Knowledge, Personal Character, Relationships and Meaning of Safety.

**Discussion:**

These results will be used to develop and test a pragmatic, accessible tool to enable providers a way to collaborate with patients for determining their personal level and type of safety involvement.

**Conclusion:**

The most ethical and responsible approach to health‐care safety is to consider every potential way for improvement. This study provides fundamental insights into the complexity of patient engagement in safety.

## 1 INTRODUCTION

The potential for harm inherent in health care has the attention of stakeholders as never before. With this knowledge, there has been a proliferation of strategies and interventions designed to improve health‐care safety. One of the strongest endorsements for the involvement of patients and families in the attempts to prevent health‐care harm has come from the World Health Organization.^1^

Health‐care safety strategies for patient involvement have been developed in Canada (eg Canadian Patient Safety Institute^2^) and internationally. However, there is limited evaluation of the adherence to, and effectiveness of these strategies, with some authors noting the lack of patient perspective, or use of evidence, in their development.^3^ Further, we could not find studies about patients’ experiences of and perspectives on safety engagement across the continuum of care during hospitalization, only on certain episodic tasks. There are also no validated tools to determine engagement preference and assess patients’ involvement in safety while in hospital. An inductive exploration of patients’ and families’ perspectives about if and how they should be involved clinically in patient safety was required.

## 2 BACKGROUND

The involvement of patients as active participants in error prevention has gained momentum in the last several years, particularly with the launch of the World Health Organization's *Patients for Patient Safety* programme.^4^ These partnerships, including others such as *Consumers Advancing Patient Safety* 5 and *Partnership for Patient Safety* 6 in the United States (US), aim to promote the voice of patients in the safety movement. However, a limited number of investigators have studied what individuals believe about participating in patient safety, and specifically at the bedside.^7^, ^8^, ^9^, ^10^, ^11^, ^12^, ^13^, ^14^ In a study of 2078 randomly selected discharged adult patients from 11 Midwest hospitals in the US, 91% agreed that patients could help prevent errors.^14^ The finding from a systematic review of generally favourable attitudes among patients to participate in safety strategies^12^ is supported by others, notably opinions from patients who cite the importance of partnership and shared responsibility.^13^, ^15^, ^16^ These overall positive attitudes, however, are qualified by several factors. First, patients are less willing to participate in challenging health‐care providers’ behaviours, such as asking staff if they have washed their hands. Rather, patients’ preference is for more traditional fact‐gathering approaches that are perceived as less confrontational.^8^, ^10^, ^12^, ^17^ Similar results were reported in a study of 491 older adults who believed their role in safety was to passively follow instructions.^11^ Perception of self‐efficacy and belief in the effectiveness of a particular strategy appear to influence the likelihood of an individual's action.^9^ Secondly, health‐care providers’ encouragement appears to favourably influence patients’ reported willingness to engage in certain safety‐related behaviours^8^, ^12^, ^17^, ^18^ which mirrors patient participation in general.^19^ Additionally, although credible evidence is lacking, it is not well understood whether the setting influences an individual's perception of the role they can or should play, varying, as example, from primary to tertiary settings.^11^, ^17^

Investigators have detailed patients’ strategies to protect themselves, often undetected by health‐care providers.^20^ Taking a family member or friend to a health‐care appointment was frequently reported across primary and ambulatory settings and included having them act as an advocate.^21^, ^22^, ^23^ Protecting oneself was expressed by giving more information to the physician in primary care settings,^22^ questioning the name of an unfamiliar medication or a change in its colour while in hospital^24^ and considering their own sense of involvement and responsibility in home settings.^25^ Mothers’ sense of vigilance over their hospitalized children and the efforts taken to “…successfully safeguard”^26^ them is poignantly described.^11^, ^27^ The vigilance undertaken by family members of patients of minority cultural and language backgrounds is noted.^28^ Finally, reports of patients’ involvement in ameliorating errors lend a strong argument for their safety involvement.^12^, ^29^, ^30^, ^31^, ^32^

Overall, there are gaps and inconsistencies in the literature, which include how safety is perceived by patients depending on the settings and across populations, the actual (vs anticipated) actions patients feel most comfortable in performing and the effect of these actions. If there is encouragement that patients have a role at the bedside in ensuring their safety, more substantial evidence is needed to determine the most appropriate and beneficial strategies for their involvement that is based on patient and family insights, not provider‐driven.

## 3 METHODS

The overall objective for this study was to gain insight into patients’ perspectives about their knowledge, comfort level and behaviours in promoting or helping their safety while receiving health care in a Canadian hospital. The primary research question was: *How do patients describe healthcare safety and what are their attitudes and beliefs about their role in promoting it while receiving care in a community hospital?* To further elucidate patient perspectives about different aspects of safety engagement, secondary research questions included: *What behaviours do patients report in ensuring their safety while receiving care in a community hospital? What enables and hinders patients’ involvement in ensuring their safety while receiving care in a community hospital? What information needs do patients report about ensuring their safety while receiving care in a community hospital? What activities do patients report are comfortable to do to ensure their safety while receiving care in a community hospital?*

### 3.1 Research design

The study was approached from the interpretative paradigm with an emphasis on describing and understanding.^33^ The study design is descriptive exploratory and it is categorized as generic qualitative research, which is defined by Caelli et al^34^ as a qualitative endeavour without being shaped by one of the known methodologies.

### 3.2 Setting

The setting was a community hospital (52 beds) in Ontario, Canada. At the time of the study, this hospital had 24 medical/surgical beds, four special care beds (level 2 ICU), 22 complex continuing care beds, two palliative care beds and outpatient ambulatory clinics.

### 3.3 Participants

The participants were adult inpatients or outpatients receiving care at the study site. To be eligible, participants had to be (a) able to speak and read English; (b) 18 years of age or older; (c) able to provide consent; and (d) medically stable as determined by the health‐care providers. Further, for the inpatient group, those who participated must have spent at least one night in hospital prior to being interviewed and were soon to be discharged. The family members were included in the interview as desired by the participant, and their comments were incorporated into the transcripts and analyses.

### 3.4 Interview tools

The open‐ended questions developed and used to garner information from participants were based on professional knowledge and common sense. The topics for some questions were informed by existing patient safety strategies^2^, ^35^ and the study site's patient information booklet, as well as common clinical processes (eg administration of medications; diagnostic testing; and staff hand washing). The questions were written at a Flesch‐Kincaid grade level 5 to reduce the need for clarification and as part of best practice to facilitate patient understanding.^36^ The demographic questions included (a) age in years; (b) gender; (c) reason for admission; (d) length of hospitalization; (e) health status; (f) previous hospitalizations; and (g) previous personal experience with adverse events in health care. All the patient information was collected from the participants only.

### 3.5 Procedure

The associated university research ethics board and the study site granted ethics approval. In the inpatient units, the nurses helped identify any eligible patients. Once patients were identified, staff approached them with a recruitment brochure to inquire if they would be interested in meeting the researcher (LD). The interviews were audio‐recorded.

### 3.6 Data management and analysis

All the data were treated as confidential, and the master participant list was kept separate from the raw data. The audio‐recordings of each interview were transcribed verbatim. Code words were created for all proper nouns and kept in a separate code sheet.

Inductive content analysis was employed for analysing the patients’ narratives to identify prominent themes and patterns.^37^ This process involved a first and second cycle coding process, wherein transcripts were coded in the first phase, and the codes were categorized into larger groupings/themes in the second phase. The family members who joined the interviews were also given a “family” code name linked to the related participant, and their statements were analysed based on the content and coded accordingly. This permitted analyses of all content as appropriate, as well as tracking of whether data were provided by a participant or family member.

### 3.7 Trustworthiness

To ensure the integrity of this research, a Model of Trustworthiness was used and considered truth value (credibility), applicability (transferability), consistency (dependability) and neutrality (confirmability).^38^, ^39^ The techniques used to ensure credibility included considerable time with each participant, as well as with a number of participants, which spanned over many months. Participants were asked their opinions about new ideas mentioned by previous participants to ensure concepts were explored in‐depth as needed. Related to transferability, the participant and setting details, as well as the rich, descriptive data from the study findings are valuable information for making informed comparisons to other contexts. Dependability was assured by accuracy of transcripts and auditable data analyses. Additionally, interviewing continued until it was determined that there were no new general themes. Regarding confirmability, an audit was not conducted, however, records (eg raw data; process notes) were maintained for every phase of the study. The lead author (LD) who conducted the interviews reflected on biases and perspectives through journaling, as well as continually discussing any concerns or reflections with her co‐investigator.

## 4 FINDINGS

**Figure 1:**
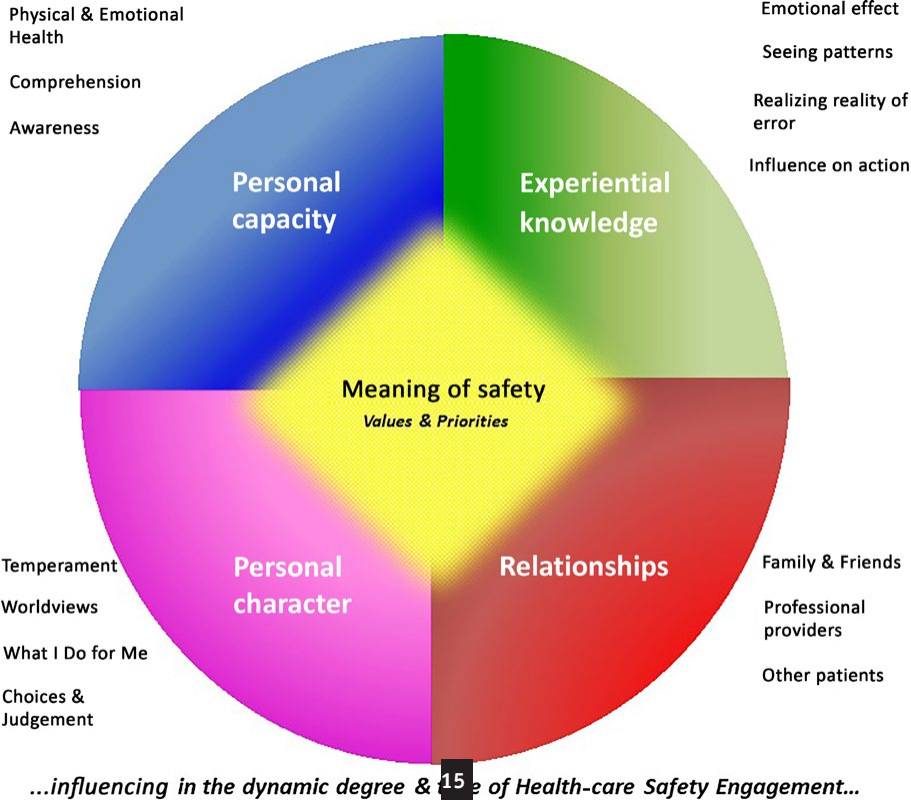
5‐facet framework to describe patient engagement in patient safety

Fourteen women and 16 men (and four of their family members) were in this study, who ranged in age from 40 to 93 years old (average age 71 years old). Eleven individuals described a health‐care error(s) (personally or via a family member). All of the participants had had previous interaction with the health‐care system for different needs, and the reasons for their current admissions were varied, including but not limited to suffering a stroke; receiving care post‐surgery that was performed at another site (eg knee surgery); pneumonia; chest pain; bone fracture; cholecystectomy; bowel surgery; bleeding ulcer; cataract surgery; complications related to congestive heart failure or chronic obstructive pulmonary disease. In describing their current and previous health‐care experiences, as well as targeted topics based on all the research questions, five main overarching themes (with subthemes) were identified: *Personal Capacity; Experiential Knowledge; Personal Character; Relationships; and Meaning of Safety*, and these were composed as a framework as defined by LoBiondo‐Wood et al.^40^ Collectively, all the themes are like the shards of coloured glass in a kaleidoscope—for each person, those facets are uniquely theirs, integrated and dynamic. Figure 1 is a visual representation of this *5‐Facet Framework for Patient Engagement in Patient Safety*. The names used herein include pseudonyms and real names.

## 5 PERSONAL CAPACITY

### 5.1 Physical and emotional health

The participants talked about the severity of their illness/injury and of resulting limitations, as well as its evolution and influence on engagement. The participants who agreed with the premise of patient involvement in patient safety qualified it by saying that illness might preclude them from being engaged. Cindy, who was asked whether patients should be responsible for protecting their safety, said, *to a certain point but I think that the hospital should be the ones that really look out for your best interests and protect you*. She further reasoned:

When the person is sick they should be taken care of and kept safe and protected…because you've got enough worries yourself when you're sick, you're under enough strain and pressure worrying about what they're going to do and how things are going to turn out.

### 5.2 Comprehension

The participants identified that individuals will differ in their capacity to understand and to remember. This type of capacity can influence if and how engagement can occur. Mildred, admitted for fluid retention, was not certain of the cause, replying, *I couldn't tell you because it's too many big words*. Conversely, Henry, having had numerous hospitalizations, stopped the interview when he heard an alarm sounding that he could not discern, to check his oxygen saturation level. When asked if she thought patients should participate to ensure safety, Maria answered, *to a certain extent,* but qualified, *I wonder how some people understand it*.

### 5.3 Awareness

Observation and awareness of hospital processes differed between individuals. In discussing staff handwashing, Arthur admitted he did not know if they had, saying, *Maybe they are, I'm just not noticing or paying attention*. Arthur's mother added, *I've seen them using that* [hand sanitizer]. In another example, Arthur's mother mentioned the hospital's gown designed with a “wash your hands” reminder. He had not paid attention to it, while his mother had, saying, *I thought that was a great idea—I notice things like that*.

## 6 EXPERIENTIAL KNOWLEDGE

### 6.1 Emotional effect

In describing their experiences (eg typically related to an error), participants revealed varying emotions and talked about feeling anger, worry or having compassion. Most often experiences of health‐care error were in the past; however, the emotional intensity was still evident. Mildred vividly recalled her anger regarding a lapse in care.

My daughter went for a mammogram and they never sent her report to her doctor. By the time *[daughter's name]* went for her check‐up, the doctor said to her did you go for a mammogram and she said yes. She was in her fourth stage of breast cancer at that point. We were all very angry.

The participants also expressed empathy and understanding about error. Otis’ experience with his mother's medication error illustrated several concepts—the upset and difficulty of seeing a family member suffer because of an oversight; the anger expressed by his family; and the ultimate resolution and understanding that errors happen. He said,

I saw how she suffered and suffered trying to do dialysis *[required due to the medication error]*. It was not a nice thing to see a family member going through. It was human error. Some of my family was going to sue and my mother said no. This was a good doctor and it fell through the cracks.

Additionally, the participants gained confidence and comfort in attaining certain experiences, while unknown situations induced trepidation.

### 6.2 Seeing patterns

For some participants, their way of understanding the health‐care environment and what might be expected of them involved looking for routines or patterns and that was afforded by time spent in other clinical settings and or the current one. By understanding the patterns or processes, this allowed for a certain control and ability to act in a seemingly vulnerable and dependent position. Sue, having had a longer hospitalization, had come to experience a certain routine and when that changed one day, she tried to make sense of it. She said,

I notice things. Normally they take your vitals around twelve o'clock at night – they didn't last night. This morning I was up early because I heard *[name]* walking in the hall, and sat down and I kept thinking they usually take my vitals before they bring my meal and they didn't today.

Aidan had had a number of prior hospitalizations and, like many, had brought his medication list to the hospital. He explained his rationale saying, *usually when I've been over before they ask me… so I always carry the medication list in my wallet*.

### 6.3 Realizing the reality of error

Participants came to appreciate the reality of error by virtue of their experiences. Several articulated a realization of the potential for error that they had not grasped or had awareness of before their experience. Their experiences brought perspective that errors could happen to them. Barb matter‐of‐factly commented that, in light of her incident, *it makes* her *[me] understand that doctors can make errors*. Henry still grappled with the realization of his medication mistake. He related, *this goes to show you that it can happen – I know humans all make mistakes, but in healthcare you've got to be extra careful*.

### 6.4 Influence on action

The participants’ experiences had varying implication on their behaviour. Of the individuals who had experienced a health‐care error, some did not feel it had significantly altered anything, while others spoke of changes. When asked if past difficulties had changed her, Paula said,

It's not so much as I don't trust them, I will question. I didn't before – it was God's word. Because that was the way I was raised. Doctor was God. You did not dare question or ask, anything. Their word was absolute. Now I will ask. And I will take a notebook and I will write down so that I have a copy of what's been said.

Ron changed physicians after his misdiagnosis, and because of the error associated with his mother's care, Otis avoided taking medications despite their indication.

Regarding future health‐care interactions, individuals dichotomized between where they had had a positive experience, and a negative or unfamiliar one. Aidan thought he would be more observant and hesitant if he returned to the site where he had had difficulty with his care. He confirmed that unfamiliar settings which had not garnered his confidence would necessitate his keen awareness. Gene, having experienced an error elsewhere, had examined the study site's website [infection rates] prior to his admission—something he would do again if going to a new setting. His wife, based on the positive experience at the study site, felt that he would be more *diligent* and would address issues as needed at new sites. Given his combined experiences, she believed he would be *a lot more capable and knowledgeable and say wait a minute* [if concerned].

## 7 PERSONAL CHARACTER

### 7.1 Temperament

Individuals revealed their personalities, sometimes apologetic for certain traits, sometimes unabashedly admitting characteristics, and often aligned with how they approached health care. Russ believed he must abide by the instructions of the providers, saying, *it's* [health‐care system] *out of my control – you've got to go with the flow, don't ask questions, just go do it*. Dan said, *I just come and go with the flow,* and was comfortable relinquishing control. Others recognized in themselves a hesitancy to act for fear it upset or was disruptive to a provider. Mary had not asked a specific question, revealing *I just don't like making waves of any kind*. Wanda, however, identified her need to *question more,* a contrast to her husband's nature. Some were selective in what they wanted to know, while others were interested in everything.

### 7.2 Worldviews

The participants disclosed different beliefs and life philosophies, detailing how these were realized in their behaviours and interactions. The participants’ worldviews, although varied, were similar in the importance they held for each individual. Peter's tenacity and resolve to get better was fuelled by his belief that you have to be determined and have positive thinking. He remarked that, *determination's the most important thing*. Similarly, Mike's worldview was captured in his words, *I always try to make it a positive experience*, which included supporting staff and encouraging an optimistic outlook.

### 7.3 What I do for me

Participants described personal strategies they used for their hospitalization, and how they coped with perceived deficits by, as example, implementing tactics from home. These safety strategies were independent of provider requests and included requesting raised bedrails at night; using a walker or cane; and consistency in wearing slippers or shoes.

Participants spoke of independently seeking and reading information (eg on the Internet). One person noted he did *a lot of research at home and here* [hospital] using a mobile device to better understand his health and procedure. Sue, however, was sceptical of searching for medical information and was *not a great believer in looking up things*…*because you can say I've got half of those things*. Another participant was not entirely sure why he had not read the 22‐page booklet about his medical procedure; however, his mother, who had read it, offered that he was *not one to sit and read*.

Medication was seen as personal and more relevant to one's control. Individuals said they would not feel comfortable asking a provider about handwashing, despite safety implications, yet had no hesitation in clarifying medications. It was suggested that medications *seems more personal to me* [patient] *and not to them* [providers]*—*handwashing is *different than asking them about something that I'm going to be taking*. In receiving in‐hospital medications, their involvement included checking the medications in varying ways and degrees of consistency (eg “sometimes” vs “always”). Strategies included asking about the medications; visual assessment for familiar cues, such as the number and colour; and taking comfort in how one was feeling as determination that there was no problem. For those who placed complete reliance on provider processes, this was based on a belief that it was not a patient responsibility and/or was due to system limitations that left no other choice.

Several individuals commented they had not engaged in any safety strategies; however, examples were identified. Dan, self‐described as accepting and laidback about his care, ultimately revealed he did check his intravenous medication. Those who believed they had done nothing exceptional but were shown how their behaviours had enabled safe care, described their actions as *automatic*. Additionally, some discounted their strategies as tactics (eg having a family member present), if it had not resulted in a need for them to be used.

The participants revealed their thoughts about being actively involved in safety. Paula, having experienced health‐care error, was emphatic and pointedly stated: patients *n‐e‐e‐d to be involved*. Aidan was also firm and pragmatic, seeing the necessity that *it has to work both ways* for overall effectiveness, which was echoed in Kevin's words, *safety is everybody's responsibility*. Wanda, equally resolute, shared, *if you're not mindful and cognizant of everything that's happening to you and around you, then you have no one to blame if someone doesn't look after you properly*. She clarified that she did not intend for patients to be *a doctor in training,* nor that it diminished the trust she felt for providers, but she could see system vulnerabilities (staff working long hours; many people and things to remember). She said, *you're the number one person that should be looking after yourself, you're your best protector*.

There were individuals who believed in safety engagement in varying lesser degrees of agreement. They saw limitations precluding their absolute involvement or had a personal belief that their role in this context should be minimal. Further, engagement and the need to assume responsibility may change depending on the circumstance as judged by the patient. Gene said, *it wasn't really necessary in this hospital but certainly in other hospitals it might be*. Otis characterized it as only to *help out*, and *only if one would like to be involved*. There were participants who did not think that patients had a role in patient safety. Their reasons included feeling that nothing needed to change, and that the role health‐care providers had and their associated responsibilities were satisfactory—this was providers’ jobs.

### 7.4 Choices and judgements

The participants made choices and judgements while in hospital. As example, they made different judgements about asking providers if they washed their hands, something that is encouraged in patient safety strategies.^2^ For some who reported being unsure if a provider had washed their hands, they had chosen not to question it or trusted that it had been done. Other rationalizations included whether they felt they knew enough about how to help ensure they receive safe care. Some participants identified that they felt they knew what they needed. Arthur declared he did not need to know more than he did because *it's supposed to be their* [provider's] *job*. His benchmark was his work and if that required 100% accuracy, he reasoned that others could achieve it. Fred, however, conceded he likely did not know enough, but was not troubled given he had no concerns. For those who would like to know more about helping ensure safe care, some qualified it with: *there comes a point where there's only so much information that you need to know and then the rest of it you don't*.

## 8 RELATIONSHIPS

### 8.1 Family and friends

In differing ways, family members taught, encouraged, advocated for and challenged each other. They were the participant's support when they were too sick, unaware, distracted or uninterested. It is not, however, to say that participants always followed what they learned from family or friends. Maria and Arthur, who described their hesitancy in asking about handwashing, could reference others they knew who behaved differently. The shared experiences with family members could also influence a participant's understanding. Wanda recounted,

I was amazed at the things I learned *[at daughter's prenatal classes]*, and it wasn't just about what happened to me, but about things that surrounded me…from that time on I became far more aware of how important I thought it was to know exactly what was going on.

When a participant's family member was present, one individual's perception was tested against another's, as well as the “checking in” with each other about the correct details of an issue or event. Additionally, there were times when a family member helped to re‐phrase a question if they thought the participant misinterpreted or did not understand. The families also seemed to have “a sense of knowing” how they needed to function and each other's roles. One gentleman counted on his family for guidance in health matters, saying, *they know what to do for me,* and equally his mother accepted that he *relies on us to let him know what's going on*. Further, family members enacted their own safety strategies. One individual explained, *I feel that sometimes he* [husband] *doesn't always ask the questions I think he should, so I'll step in and ask them for him*.

### 8.2 Professional providers

The participants commented on interactions with health‐care professionals, including their expectations of providers and their efforts to facilitate that relationship (eg such as through the use of humour). They defended health‐care providers [past or current] and protected that relationship, even if mistakes in care had been made. They made allowances for, as example, late medications or delayed call bell response, appreciating providers are busy. If something had not yet been discussed or arranged, they made assumptions and trusted it would be managed. The participants who identified a medical error often tried to minimize the event. Ross, who needed to be hospitalized for a past medication error, declared of the incident, *that was just an isolated little case there, that was all*. While Barb remained steadfast that her provider was *a good doctor,* the paradox of her description of the incident revealed deeper, conflicted feelings as she admitted, *but I do feel like what my doctor missed was a bit major*.

When participants described negative attributes about an interaction with a health‐care provider it often centred on how they were made to feel. One participant recounted a past experience at another site, revealing that while his doctor was amazing, he felt that *the staff didn't really care one way or the other about you personally*. His wife was able to contrast between the positive interactions at the study site vs her husband's past negative experience. She articulated:

I think that hands on attention *[at study site]* and the interest in how the patient was feeling – ‘are you worried’ and ‘everything's going to be alright’. It makes a huge difference—you're a person versus a case.

Sarah said of the providers, *they seem to care about you – and when we feel that, we feel stronger because we feel more secure, safer*.

The participants identified talking with, learning from and working with health‐care providers as safety strategies. The participants’ involvement included alerting staff if they noticed anything amiss; requesting a medication before a condition exacerbated; and talking with providers preoperatively about issues that they thought staff should know. One participant detailed tracking his intake and output to not only help staff, but *to make sure that they weren't giving me too much medication*. Ilah felt it important that providers *should accept it* [involvement].

### 8.3 Other patients

As participants described their experiences, they included roommates. They shared how roommates often helped each other, such as seeking medical help when one was in need. Peter, having fallen, acknowledged the good fortune that his roommate was nearby saying, *lucky enough there was another gentleman in my room and he called for help*. Ilah also spoke about a previous time when her roommate had acted on her behalf by yelling to get help.

## 9 MEANING OF SAFETY

### 9.1 Values and priorities

The participants had distinct viewpoints of safe care and what “feeling safe” meant. There were times when participants struggled to find the words, likening it more to an elusive “feeling” and something that they knew they received but could not articulate. Mobility and fall prevention, the environment, and insightful, attentive staff, figured prominently in their responses. While practical considerations were provided, more conceptual ideas of safe care were also described, as when Dan talked about it being *fair* and *honest*. Table 1 provides examples of the participants’ views about the important elements of safe care.

**Table 1:** Examples of how individuals define safe care and feeling safe

In the words of participants: what safe care and feeling safe is about & should be…
Elusive, hard to describe… I do believe I get safe care *[at study site]* except for the odd little thing. I know I have safe care – I do. I feel it. I don't know what to say about that. I'd like to know they got safe care, I assume they do. It's just something I take for granted.
Mobility & fall prevention… Providing things when you need to move about safely – like giving you a walker if you need to walk somewhere or wheelchair and they make sure it fits my body comfortably so I can get around better. They take me to the bathroom if I need it and stand by so I don't get up and fall. They make sure you're sitting down safely before they leave the room and…or in bed or whatever. I do feel safe here. I guess because I can get around. To make sure, if they're so sick they don't know what they're doing, for themselves, that they're not going to be able to fall or fall out of bed or if they are trying to get out of bed then, that's when they're going to get out of bed anyway because you can't be everywhere at once. If you can't keep them in the bed at all then you take all precautions that that one's not making the decision on their own to get out of bed. And also if they needed to be strapped in then they are, it's for their protection and the staff protection to do it. I think when you're unsteady on your feet you kind of need that extra little hand to help you.
Rushing feels unsafe… *[Anything that made you feel unsafe]* Just the rush. It's like I've *[staff]* got to get this job done because I've got another really important job I've got to get to. I'm in a hurry. *[worry miss something]* I do. We have to streamline everything.
Away from stress of roommate… Away from a tyrant *[roommate]*.
Infection control… You want to make sure that you're not going to give any germs if you have them to somebody else. There could be times when they *[staff]* should wear a mask too maybe.
Teaching (or not)… What the nurses teach us. How to sit down, how to get up, always back up to a chair till you feel it at the back of your legs and then you can sit down. If somebody doesn't know what they're doing…they can tell you if you don't do this something like this might happen. *[Would teaching on medication make you feel safer]* Yes, I think it would. Yes – they *[patients]* will know what is required of them to make sure that they're safety is looked after. Make sure that they can. Yes, that's for sure. Especially the physio part. No, I've been in hospitals enough to know what goes on.
Everyone follows safety standard… The hospital treating you was ensuring your safety by having the best trained or qualified people to look after you, to ensure that they wash their hands, to ensure that they're kept up on the latest methods, to ensure that other hospital staff such as, cleaners, food, volunteers, all maintain that safety standard too and that cleanliness factor of cleaning their hands before they come in. *[Unsafe]* The non‐caring nurses that administered it *[treatment at another site],* that didn't follow protocol, I'd call that unsafe care. Very scary.
Environment… That if they're mopping the floor they put the signs out and they warn you. Or if they're making your bed, they make sure they haven't got it up too high that you can't get into it. That they have the furniture arranged so that you're not going to fall over it. Stuff is placed so you can get at it. I'm in a safe and clean environment which means a lot to me. *[Unsafe]* These floors are very slippery. I have to wear my slippers all the time. But they force me to wear my slippers anyways. It's your environment, anything on the floor, like if I look around and say see something on the floor and anything's not going to fall off. Your total environment, just that, something's not going to be some kind of a little accident happening or something. Watching your cords. I think that's the big one there, the clean *[hospital]*. The only that comes to my mind is the smaller area, make sure it's not cluttered to that if an emergency comes up that you have to remove the person from the room, that you can get the bed out safely. *[clean environment part of safe care]* Definitely. Yes. I like things being clean. I can't stand anything dirty. When I see things dusty it makes me feel like I'm not breathing well.
Providers’ responsibility… A nurse's responsibility to make sure that her patients are getting better. The responsibility seems to stem back to the nurses…proper care from them. That they do their part. Teamwork. Safe care is you're in the hospital, and you've got the nurses and the medical people looking after you, I figure that's safe care. That they're doing what they should do to help me. I'm in the hands of them.
Not threatened… I don't feel threatened by anything.
Medication administration… Your medications should be looked at but I don't know maybe they do look, the nurse who brings it to you. Getting the right drugs, the nurse checking that the drugs are the ones you're supposed to get, washing her hands before she gives them to you.
Insightful, reliable & attentive staff… Being there when you need them. I feel that they should be here, like if you ring the buzzer and you're in a lot of pain and you know that you're afraid to get up to do things on your own, that's my only concern, that they would come and do it, for you, help you. Be prompt, and that if you need them, to get here as soon as possible. I don't think you should leave the patient alone so much. I know they've cut back but. But these horror stories about retirement homes and…terrible. Just awful and they're right here in our own community. They assessed each person whether they were nervous or they were apprehensive or they were relaxed. I think they should look in on you more often than they do. They do have a bell if you need it but you have to remember that there's a bell there or you have to be awake enough to know “yes” you've got to press the bell. That's where the problem is. And sometimes…you can't see how sick I am, I can't see how sick you are. I don't know how you feel, you don't know how I feel. That's hard. They're there when you want them—I think that's the bottom line. Being able to have somebody respond when you ring your bell quickly.
Effective communication… Yes. Exactly. He *[anaesthesiologist]* was very reassuring, every time I saw him, he would touch me on the shoulder – “you ok now, it's just me,” you know and I had my eyes shut and he said, “it's just me again and I'm going to be…” and “just me.” So, he made me feel relaxed. Even the surgeon who I'd not met, he was very good as well. He introduced himself, he asked me if I had any questions. And he explained the procedure all over again, told me what we were going to do, told me that I was to participate, and that was fine *[she chuckles] –* I said am I going to be out for this and he said no. And I said well that's good. And both the anesthesiologist and the doctor said that they wanted me to participate, and I felt good about that *[having awareness]*. *[feeling unsafe]* Yes, when the fire alarm went off. The first time it happened, they did say we'll close the door. But last night, nothing happened, it just kept on and on – soon the buzzers stopped and don't know if it was a fire or, somebody broke in or…nobody came in the room even. People that you could ask questions.
Fair, honest, nice… Well, you don't hear guns going off or fire trucks pulling up. I don't know, just everything is fair and honest and nice, so you feel safe. Not going to come in and steal your stuff or anything.
Being mistreated… *[safe care] Like* being mistreated, you mean?
Alerting staff… Not doing what you shouldn't do by yourself without letting, you should always let the nurses let them know if you are going to be doing something, if it's something you shouldn't be doing by yourself. Unless you've already agreed, ok, it's fine I can do this by myself. But I like the idea there's bells all over. If you do get into something you can call for a nurse.
Everyone's role… Everyone, everyone being aware.
Multifaceted… I think there would be maybe two or three different scenarios that should come into play. Make sure that the room is not cluttered, and stuff like that. The medication should be, definitely looked at properly. And also whether that patient is demanding because they are hurting in pain so much that they're becoming an annoying, so therefore it works on one's nerves, so you have to keep in mind this is not that person's right make‐up. There's safety for all, not only for the patient but for the nurse, the doctor, the staff person.
Common sense… *[Not getting safe care]* Sometimes I think common sense has been cancelled.
Small town‐feel… I feel extremely secure here. Extremely secure. I guess I'm looking at I live in a small town, I don't live in *[name of large city]* anymore. But you see forced entrances into hospitals, and whatnot. We don't think about that here.
Caring… They *[staff at study site]* don't let you go off on your own, walking around the hospital if you're not able to do it and they're here looking after you 24 hours a day. And they're always on call. *[family member adds]* In the real sense of the word, rather than just making a buck. The nurses themselves, they do all they can to help you. They do their best.
Reinforcement and reminders… They provide well, like for you to call for help if you need it and they tell you if you need anything at all call me, every night. So much reinforcement *[pre‐procedure of what will happen]*. Somebody in a wheelchair will try and stand up, they say “oh no, you have to sit down” and that's just a constant thing, they do that all the time. And just the way they set people in a wheelchair for an example. You know, “be careful, we have to do this, we have do that” and then they always put brakes on when they stop and stuff like that. Being a non‐professional may do it sometimes and not others, where they are constant with it.
Being aware… It's like anywhere else, you've *[patient]* got to keep your eyes open and your ears.
Rights… I know we *[patients]* have rights. If they think they aren't being looked after safely I believe there's someone that they should be able to go to or report to or discuss it with, certainly.

The participants’ answers about safe care were centred on the providers and health system, and viewpoints on the importance of patient engagement were not foremost. The definition of safe care was not typically inclusive of a patient's role. They reflected on how things are done to them, as recipients of all that transpires around them (for better or for worse)—a dependency. The real or perceived limitations for their involvement, or the belief that anything aligned with health care was beyond their responsibility, necessitated having *faith* in others. As such, integral to their thinking about safe care was trust. Sarah's eloquent wording was at the core of what so many indirectly implied about the Meaning of Safety: *we trust our nurses, we trust the doctors – we have to, they have the knowledge*.

## 10 DISCUSSION

Given the complex intricacies of patient engagement in patient safety, it is not a straightforward issue; we cannot simply say patients should or should not be engaged as partners in their safety. The 5‐Facet Framework for Patient Engagement in Patient Safety developed from this study is a way to conceptualize the components one must consider when engaging patients in patient safety at the bedside. A particular facet (or facets) will loom larger and their influence be more significant for one patient than for another. This was seen in the way participants emphasized certain issues, as evidenced in the time they spent talking about them, reiterating them, and in the power of their speech. Unlike others (eg Carman et al^41^), engagement is not seen as being on a continuum, but rather as much more dynamic than linear. A number of elements could influence what engagement looked like for a patient at any given time. As example, previous exposure to clinical interactions and or experience with error did not necessarily equate to an individual believing or wanting to be engaged. It is with this in mind that we must approach the dialogue about and assessment of patient engagement in safety, and—given its potentially dynamic nature—with a regularity similar to taking a vital sign.

It was revealed that every individual described a safety‐related activity they engaged in, whether they realized it as such or not. This is supported by Martin et al,^42^ who found that participants did not always identify certain actions as being related to safety. Similarly, Pinto et al^43^ reported that patients indicated they would have taken action if something was wrong even without the study intervention. Bishop^44^ described patients taking notes or having an advocate present as a safeguard. Other investigators have also identified that patients and family members (ie parents of patients) act in ways to safeguard themselves or another.^45^, ^46^

The participants based decisions on advice from family members and relied on them to, as example, bring medications or help decipher health‐care information. For many, it seemed a purposeful strategy. In this way, the family member could act as a second safety check. The results of other studies support this finding, such as the research by Rainey et al^47^ of seven family members (and 13 patients) and their vigilance. Additionally, parents with a sick child described actions they take to safeguard the child, including advocating and constant surveillance.^46^ Collectively, this evidence suggests that patients, as well as family members, are engaged in safety in their own ways.

An unusual finding of this study was that, despite individuals believing patients need to be engaged in safety, most expressed comfort with their current knowledge and understanding of safety. Further, although some admitted they probably did not know enough, they were satisfied with what that they knew. It may be that participants had difficulty articulating what information they needed, similar to participants in the study by Martin et al.^42^

Safety is principally seen as the responsibility of providers. Martin et al^42^ reported that, of the 25 patients they interviewed, safety was regarded as the purview of the provider, while the participants in Walters’ study, said that it [safety] should not be a patient's obligation.^48^ The participants in this study identified limitations to having an equal partnership, including knowledge, degree of physical and emotional wellness (as others have reported^42^, ^48^), or a fundamental belief that it simply is the professional's obligation. One can also make a general inference that there are safety elements that patients see as the sole responsibility of the health‐care professional by considering results from investigators who have examined specific clinical issues, such as patients’ [negative] attitude towards asking providers about handwashing.^49^ If patient engagement in safety is to be standardized, engagement limitations will need to be addressed (as possible), and a shift in thinking about the patient role will be required of some patients. Further, and as seen in other studies as well as this study, is the trustfulness and sensitivity patients have of their provider relationship, which must also be balanced.^18^, ^50^

Involving patients in research who wish to be, specifically in patient‐identified engagement safety strategies, is needed. We have not purposely designed assessment strategies to identify patient‐identified tactics or how they can be enhanced and encouraged, as feasible and appropriate. Improved information and communication about safety processes, and the rationale as to why certain actions are needed, as opposed to telling patients to do something, might engage them in a different, more effective way. In sum, and most importantly, discerning patient preferences as to how they see their engagement must be the goal of future work.

### 10.1 Study strengths and limitations

This study has several strengths. First, it is believed to be one of the first Canadian studies occurring in a community hospital that was designed using an interpretative approach to explore patient and family member perspectives and behaviours about their active participation in health‐care harm prevention across the spectrum of care. Other Canadian investigators have concentrated on specific processes (eg medication process^51^) or employed focus group methods for examining patient perceptions of safety, the influence of providers on patient involvement and strategies for improving engagement.^18^ Further, there was benefit in asking individuals not only what they thought or believed about participation in patient safety, but also if they would and had taken safety actions.

It is conceded that the study has limitations. One could challenge that this group of participants was not diverse, limited by the catchment area that is served by the hospital. It is offered, however, that the 5‐Facet Framework, and specifically *Personal Character* and *Meaning of Safety* themes, is aligned with these elements and are facilitative in considering and addressing where there may be differences and distinctions in this regard.

As a necessary initial step and in keeping with the study questions, the findings of this study have been presented as a 5‐Facet Framework to illuminate and describe important aspects for patient engagement in safety as expressed by patients. There was no attempt to formally characterize or delineate the specific relationships between facets, other than to generally acknowledge they were uniquely integrated for each person. While this may be a potential limitation, the utility lies in the fact that this is a building block for future work, specifically how this framework can shape the development of an assessment/evaluation tool about patient engagement in safety to be used in clinical settings.

## 11 CONCLUSION

While advocates have promoted that, “If the focus on patient safety doesn't begin with, and include the patient a valuable piece of the health‐care process is lost,”^16^ little research has been conducted to determine what patients think about having a role to ensure safety at the bedside. This study was about how patients describe their attitudes about their role at the bedside in partnering with providers to prevent harm. Based on 30 patient face‐to‐face interviews, a parsimonious 5‐Facet Framework was developed. It suggests that there are dynamic, multifaceted reasons, beliefs and circumstances why patients want and engage in safety. The future work for researchers, policymakers, providers, patients and patient advocates must be to focus on assessing what is right for each patient—some will want a more passive role; some will want active involvement. The key is talking with patients about safety throughout their care experiences, about what they see and do to promote their own safety, and to build on those approaches.

## CONFLICT OF INTEREST

None of the authors have any conflict of interest to declare.
